# Symptoms of depression and anxiety among health care workers during COVID-19 pandemia in latvia: A cohort study

**DOI:** 10.1192/j.eurpsy.2021.708

**Published:** 2021-08-13

**Authors:** L. Valaine, A. Ancans, L. Logina, R. Beskrovniijs, L. Bubko, G. Ancane

**Affiliations:** Department Of Psychosomatic Medicine And Psychotherapy, Riga Stradiņš University, Riga, Latvia

**Keywords:** physicians mental health, health care workers, Depression, Anxiety

## Abstract

**Introduction:**

Studies from the beginning of 2020 show that symptoms of depression and anxiety are increasing among health care workers. It is important to assess the dynamics of health care workers mental health.

**Objectives:**

To assess the dynamic of symptoms of depression and anxiety among health care workers over a 3-month period during the COVID-19 pandemia in Latvia.

**Methods:**

A longitudinal cohort study of symptoms of depression and anxiety in the population of physicians, physician assistants and nurses in Latvia during the COVID-19 pandemia. Symptoms of depression were assessed using the Patient Health Questionnaire-9 (PHQ-9) scale, symptoms of anxiety were assessed using the General Anxiety Disorder (GAD-7) scale, cut-off score for both scales was 10. Initial data was collected on April-May 2020 with a 3 month follow-up.. Data was analyzed using SPSS- Related-Samples McNemar test.

**Results:**

348 physicians were initially included (women 83,9%, mean age 45,17±14,02) and 376 physicians assistants and nurses (women 88,2%, mean age 39,99±12,97). After the 3-month follow up 189 physicians (women 88,40%, mean age 45,01±13,57) and 141 physicians assistants and nurses were left (women 88,00%, mean age 39,96 ±12,59). During the 3 months symptoms of depression among physicians rose from 26,80% (n=94) to 27,5% (n=52), symptoms of anxiety from 17,70% (n=62) to 20,6% (n=39). Depression symptoms among physician assistants and nurses dropped from 25,50% (n=96) to 23,9% (n=34), symptoms of anxiety stayed almost the same 18,20% (n=68) to 18,30% (n=26). Symptoms of depression among physicians changed from 26,80% (n=94) to 27,5% (n=52), symptoms of anxiety from 17,70% (n=62) to 20,6% (n=39), changes were not statistically significant (p=0,281; p=0,725). Symptoms of depression among physician assistants and nurses changed from 25,50% (n=96) to 23,9% (n=34), symptoms of anxiety from 18,20% (n=68) to 18,30% (n=26), changes were not statistically significant (p=0,405; p=0,664).

**Conclusions:**

No change in the dynamics of symptoms of depression and anxiety among health care workers over a 3-month period during the COVID-19 pandemia in Latvia was observed.

